# Beta-hydroxy-beta-methyl butyrate supplementation in critically ill patients: a systematic review and meta-analysis of randomized controlled trials

**DOI:** 10.3389/fnut.2025.1505797

**Published:** 2025-01-23

**Authors:** Yu Ren, Ya-Bei Gao, Da-Xing Yu, Hui-Bin Huang

**Affiliations:** ^1^Department of Emergency, Fuxing Hospital of Capital Medical University, Beijing, China; ^2^Department of Critical Care Medicine, Beijing Fengtai Hospital of Traditional Chinese and Western Medicine, Beijing, China; ^3^Department of Critical Care Medicine, Guang’anmen Hospital, China Academy of Chinese Medical Sciences, Beijing, China

**Keywords:** beta-hydroxy-beta-methyl butyrate, critical illness, muscle mass, mortality, meta-analysis

## Abstract

**Background:**

Beta-hydroxy-beta-methylbutyrate (HMB) is beneficial for restoring muscle mass. However, the evidence supporting its use in critically ill patients remains unclear. We conducted a systematic review and meta-analysis of HMB in this population to ascertain its effects.

**Methods:**

We searched PubMed, Embase, China National Knowledge Infrastructure, Wanfang, and the Cochrane database for articles focusing on adult patients receiving HMB compared to controls. The primary outcome was mortality. To explore potential heterogeneity, we assessed study quality and performed subgroup analysis, sensitivity analysis, and quality of evidence.

**Results:**

Nine randomized controlled trials were included. There were some differences in the study design, HMB protocols, and muscle measurements among these trials. Overall, there were no significant differences in mortality between the HMB and the control groups (risk ratio = 0.96; 95% CI, 0.44–2.08; *P* = 0.92). This finding was confirmed by the subgroup and sensitivity analyzes. Patients in the HMB group had similar durations of MV [mean difference (MD), –0.40; 95% CI, –0.91 to 0.12; *P* = 0.13], ICU stay (MD, –0.61 days; 95% CI, –3.59 to 2.38; *P* = 0.69), and hospital stay (MD, 1.52 days; 95% CI, –1.18 to 4.22; *P* = 0.27). In addition, HMB did not affect changes in body weight (*P* = 0.53), body mass index (*P* = 0.56), or quadriceps thickness (*P* = 0.74). The outcomes of changes in skeletal muscle area (*P* = 0.95) and muscle loss (*P* = 0.16) were similar between the two groups.

**Conclusion:**

Beta-hydroxy-beta-methylbutyrate (HMB) did not improve the mortality or other clinical outcomes in critically ill patients. This may be because of the different HMB strategies used in the included trials. Our findings provide insights into future research designs that explore the clinical efficacy of HMB in this patient population.

## Highlights

• HMB did not improve mortality or other clinical outcomes, nor did it affect muscle volume in ICU patients. These negative results may be attributed to different HMB strategies, including rehabilitation exercises, HMB dosage, timing of outcome assessment, and nutritional adequacy.
• Larger, adequately powered RCTs with rigorous definitions and designs are warranted to confirm our results.

## Introduction

Critically ill patients often experience significant stresses (1). This leads to hypercatabolism, which can lead to various complications including skeletal muscle loss (1, 2). Research has shown that the rectus femoris cross-sectional area decreases at a rate of 1–2% per day in intensive care unit (ICU) patients, with muscle protein loss reaching nearly 20% on day 10 after ICU admission (3). Notably, patients with multiple organ failure experience greater muscle decrease than those with single organ failure (3). Muscle wasting is caused by a variety of factors, including infection, corticosteroid administration, immobility, mechanical ventilation (MV), sedation, and neuromuscular blocking agents (4, 5). Additionally, sepsis can promote upregulation of gene expression associated with muscle protein degradation (6). Loss of skeletal muscle protein can lead to ICU-acquired weakness, diaphragm dysfunction, and ventilator dependence (7, 8). This results in increased ICU stay, MV duration, and mortality (7, 9). However, current strategies to reduce muscle atrophy, such as infection control, increased protein supplementation, and pharmacological treatments, are not so effective.

In recent years, beta-hydroxy-beta-methylbutyrate (HMB) has been found to mitigate muscle loss and promote muscle synthesis (10, 11). HMB is a metabolic derivative of leucine and is an effective regulator of muscle protein turnover (12). It can strongly induce muscle protein synthesis and inhibit proteasomal degradation in skeletal muscles (13). HMB has been shown to safely reduce sarcopenia in elderly patients and help healthy individuals recover from exercise-induced muscle damage (14–16). However, there is little evidence of its use in critically ill patients. A recent umbrella review (17) included 15 meta-analyses, nine of which were in the elderly, 2 in oncology, and 2 in various clinical scenarios. In a previous meta-analysis targeting clinical populations, 15 studies involving 2,137 participants with muscle weakness and loss (i.e., elderly postoperative patients, malnourished individuals, and patients with cancer) suggested that either HMB alone or supplements containing HMB could increase muscle mass and strength, although the effects were small (18). However, this meta-analysis included only two studies that involved critically ill patients (18), thus limiting the ability to draw relevant conclusions.

Recently, several published studies have shown that HMB may be beneficial for ICU patients in terms of nutritional status or nitrogen balance (19–21). However, there is limited evidence that HMB improves muscle catabolism and patient-centered clinical outcomes (22, 23). Therefore, with the power of meta-analysis, we aimed to conduct a meta-analysis to investigate whether HMB supplementation could be beneficial for muscle maintenance and clinically important outcomes in critically ill patients.

## Materials and methods

This systematic review and meta-analysis was conducted according to the Preferred Reporting Items for Systematic Reviews and Meta-analysis and Cochrane Collaboration guidelines (Supplementary Data Sheet) (24).

### Search strategy and selection criteria

Two authors (Y-R and Y-BG) independently performed a computerized literature search from inception to May 15, 2024, using PubMed, Embase, and Cochrane Library. The search strategy included medical subject headings and free text terms (HGM OR beta-hydroxy-beta-methylbutyrate OR hydroxy methylbutyrate OR) AND (critically ill OR critical care OR intensive care) without language restrictions. The details of the full search strategy are summarized in Supplementary Data Sheet. The recruited articles were also examined in any of the eligible studies.

The inclusion criteria were as follows: (1) population: critically ill patients aged 18 years or older; (2) intervention: HMB was used alone or in combination with other supplements, regardless of dose, route of administration, treatment course, and exercise (HMB group); (3) comparators: placebo or other HMB-free supplements (control group); (4) design: randomized controlled trials (RCTs); and (5) outcomes: studies reporting any efficacy and safety outcomes as defined by each author of the included studies.

### Data extraction and outcomes

Two authors independently extracted the relevant data from the included RCTs. The data can be extracted from tables, figures, or text. These variables included study characteristics (first author’s name, year of publication, study design, and country), patient characteristics (age, gender, patient population, disease severity, and body mass index), HGM and control protocols, and predefined outcomes.

The primary outcome was all-cause mortality. Secondary outcomes included the duration of mechanical ventilation (MV), length of stay in the ICU or hospital, muscle measures (i.e., muscle thickness or area changes, defined by each author), and adverse events.

### Quality assessment

Y-R and Y-BG independently evaluated the quality of each included RCT using the Cochrane Risk of Bias tool (version 2) (25). Publication bias was evaluated using visual inspection funnel plots when ten or more studies were included. We used the Grading of Recommendations Assessment, Development, and Evaluation (GRADE) system to evaluate the quality of evidence (26). Disagreements between the two authors were resolved by consulting with a third author (H-BH).

### Statistical analysis

The results from all relevant studies were combined to estimate pooled odds ratios (ORs) and associated 95% confidence intervals (CIs) for dichotomous outcomes. For continuous outcomes, we estimated the mean differences (MD) and 95% CIs as effective results. For studies that reported the median with an accompanying interquartile range (IQR) but not standard deviations (SD), we estimated the mean from the median and SD from the IQR based on the methods described in the Cochrane Handbook (27).

To analyze each predefined outcome, we conducted a meta-analyses of relevant trials. Meta-analyses were conducted when at least two studies could be pooled. To test the robustness of the outcomes and explore potential influencing factors, we performed sensitivity analyses to identify the influence of each study on the overall pooled estimate of the outcome of interest. Additionally, subgroup analyses were conducted separately by pooling studies based on (1) exercise: with or without; (2) location: Asia and non-Asia; (3) patient population: specific ICU patients or mixed ICU patients; (4) route of intake: oral or tube feeding; and (5) double-blind or single-blind study design.

The *I*^2^ statistic was used to test for heterogeneity, with values of *I*^2^ < 50% and *I*^2^ > 50% indicating low and high heterogeneity, respectively. A fixed-effects model was used when *I*^2^ > 50%, and a random-effects model was used when *I*^2^ < 50%, using the Mantel-Haenszel method (28). The significance level for *P*-values was set at 0.05. Review Manager (version 5.4) was used for all analyses.

## Results

### Searching results

**FIGURE 1:**
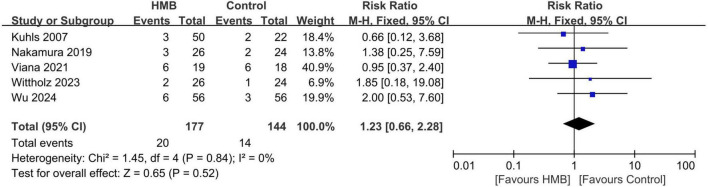
Flow chart of literature selection.

The search strategy identified 181 records from databases and additional searches. After removing duplicates, 117 records were available for title and abstract screening. Of these, 15 were retrieved for full-text screenings, with nine RCTs eligible for inclusion in the final analyses (19–23, 29–32) (Figure 1).

### Study characteristics

Tables 1, 2 present the main characteristics and study strategies of the included RCTs. These trials were published from 2006 to 2024, with study durations ranging from 999 weeks to 1 year. 99 participants were analyzed, 99 in the HMB group and 99 in the control group (19–23, 29–32). The included studies, except Wu et al. (32), were all single-center studies. These trials focused on unselected ICU populations (23, 29, 31, 32), severe trauma (20, 22), major surgery (30), and severe brain injuries (19). Five of the included RCTs used HMB as a single supplement (19, 21–23, 32), two used HMB in combination with arginine and glutamine (29, 30), and the remaining two used both HMB alone and the HMB combination formula (19, 21, 31). All studies provided 3g/day of HMB (1.5 g, twice a day). Three trials conducted exercises with HMB interventions including early electrical muscle stimulation (30), early rehabilitation (31), and resistance training (32). All studies reported on follow-ups, with the timing of outcome assessment varying between days 4 and 3 months post-intervention. The details in HMB strategies are summarized in Supplementary Data Sheet.

**TABLE 1:** Characteristics of the included studies.

References	Country	Population	Design	*N*		Age (year)		Gender (%)		Primary outcome	Risk of bias
				**HMB**	**Ctrl**	**HMB**	**Ctrl**	**HMB**	**Ctrl**		
Hsieh et al. (21)	China	COPD with MV	SC, SB	18	16	78.8	78.3	NR	NR	Anti-inflammatory effect and PF	Unclear
Kuhls et al. (20)	USA	Trauma with MV	SC, DB	50	22	38	37	72	63.6	Nitrogen balance	Low
Meng et al. (19)	China	Severe brain injury	SC, UB	28	28	52.9	52.2	64.3	67.9	Nutritional status	Unclear
Nakamura et al. (29)	Japan	Critical illness	SC, SB	45	43	69.4	72.4	60	62.8	Muscle loss	High
Norouzi et al. (30)	Iran	Heart surgery	SC, DB	30	30	59	55	70	50	Myocardial biomarkers	Unclear
Supinski et al. (31)	USA	MV patients	SC, DB	36	37	59.5	54.1	NR	NR	Muscle strength	Low
Viana et al. (23)	Switzerland	Critical illness	SC, DB	19	18	64.9	64.9	76.3	76.3	Muscle loss	Low
Wittholz et al. (22)	Australia	Severe trauma	SC, DB	26	24	52	49.5	69.2	70.8	Feasibility of administering HMB	Unclear
Wu et al. (32)	China	MICU patients	MC, SB	56	56	61.3	60.1	80.4	76.8	SPPB and 6WMD	High

COPD, chronic obstructive pulmonary disease; Ctrl, control; DB, double-blind; EN, enteral nutrition; HMB, beta-hydroxy-beta-methyl butyrate; ITT, intention to treat; ICU, intensive care unit; MC, multicentre; MICU, medical intensive care unit; MV, mechanical ventilation; NR, not reported; SB, single-blind; SC, single-center; SPPB, short physical performance battery; UB, unblind; 6WMD, six-minute walking distance.

**TABLE 2:** Study strategies of the included randomized controlled trials (RCTs).

References	Feeding type	Nutrition protocol Calories; protein	Intervention	Control	Exercise	Timing of evaluation	Muscle measure
Hsieh et al. (21)	Nasogastric feeding	NR	HMB 3 g (2 × 1.5g doses/d);*n* = 18	Usual care; *n* = 16	NR	7, 14 days	NR
Kuhls et al. (20)	Tube feeding	25 kcal/kg/d; 1.5 g/kg/d	HMB 3 g (2 × 1.5g doses/d);*n* = 28	Isonitrogenous control	NR	3 months	NR
			C-HMB 3g (2 × 1.5 g doses/d);*n* = 22				
Meng et al. (19)	Tube feeding	NR	HMB 3 g (2 × 1.5 g doses/d);*n* = 28	Usual care; *n* = 28	NR	10 days	CT
Nakamura et al. (29)	Tube feeding	20–30 kcal/kg/d	C-HMB 3 g (2 × 1.5 g doses/d);*n* = 45	Usual care; *n* = 43	EMS	30 days	NR
Norouzi et al. (30)	Oral	NR	C-HMB 3 g (2 × 1.5 g doses/d);*n* = 45	Isonitrogenous control	NR	10 days	US
				With amino acid; *n* = 30			
Supinski et al. (31)	Tube feeding	NR	HMB 3 g (2 × 1.5 g doses/d);*n* = 18	Isonitrogenous control	ER	4, 15 days	US
			C-HMB 3 g (2 × 1.5 g doses/d);*n* = 18	With amino acid; *n* = 37			
Viana et al. (23)	Tube feeding	IC-guided EN; 1.2–1.5 g/kg/d	HMB 3 g (2 × 1.5 g doses/d);*n* = 18	Usual care; *n* = 30	NR	1,7,14,21, 28, 90 days	NR
Wittholz et al. (22)	Tube feeding/oral	25 Kcal/kg/d; 1.2–2.0 g/kg/d	HMB 3 g (2 × 1.5 g doses/d);*n* = 26	Isonitrogenous control	NR	ICU, hospital	US
				With amino acid; *n* = 24			
Wu et al. (32)	Tube feeding/oral	20–25 Kcal/kg/d; 1.2–2.0 g/kg/d	HMB 3 g (2 × 1.5 g doses/d);*n* = 28	Usual care; *n* = 28	RT	7 days, discharge	NR
			RT + HMB 3 g (2 × 1.5 g doses/d);*n* = 28	RT + usual care: *n* = 28			

C-HMB, HMB combined with other supplements, such as arginine, or glutamine; CT, computed tomography; EMS, electrical muscle stimulation; HMB, beta-hydroxy-beta-methyl butyrate; RT, resistance training; ICU, intensive care unit; IC, indirect calorimetry;NR, not report; US, ultrasound.

### Quality assessment

We evaluated the risk of bias in the included studies using the Cochrane risk of bias tool for RCTs (Supplementary Data Sheet). The risk of bias in the RCTs was low in all critical domains. The assessment of publication bias using visually inspected funnel plots showed no potential publication bias in the included studies (Supplementary Data Sheet). Using the GRADE methodology, we rated the evidence for pooled data for intubation rate, mortality, and ICU stay as moderate, moderate, and very low, respectively (Supplementary Data Sheet).

### Primary outcome

**FIGURE 2:**
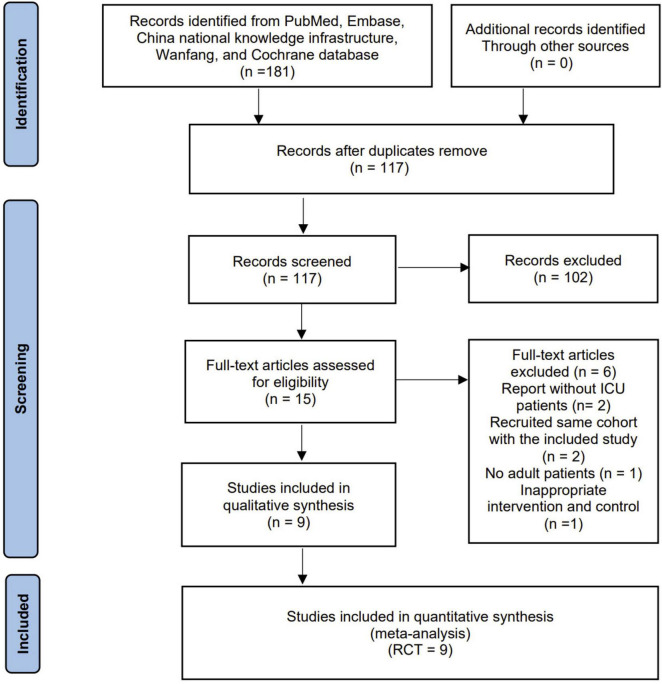
Forest plots of the beta-hydroxy-beta-methylbutyrate on mortality rate in critically ill patients.

All-cause mortality was reported in five RCTs (20, 22, 23, 29, 32). We found no significant difference in mortality between the two groups (RR = 0.96; 95% CI, 0.44 to 2.08; *I*^2^ = 0%, *P* = 0.92) (Figure 2). To investigate the sources of heterogeneity, we performed stratified analyses based on predefined main study characteristics and clinical conditions. Excluding any single study from the sensitivity analysis showed results similar to the overall combined OR (*P*-values ranged from 0.37 to 0.93, with all *I*^2^ = 0%). Subgroup analyses were also conducted. These results suggested similar mortality risks when studies with predefined characteristics were pooled (Table 3).

**TABLE 3:** Subgroup analyzes of the effect of beta−hydroxy−beta−methylbutyrate (HMB) on mortality in critically ill patients.

Study characteristics	Studies number	Patient number	Event in HMB group	Event in control group	Risk ratio (95 % CI)	*I* ^2^	*p*
All included studies	5	321	20 of 177 (11.3%)	14 of 144 (9.7%)	1.23 (0.66, 2.28)	0%	0.52
Exercise with exercise	2	162	9 of 82 (11.0%)	5 of 80 (6.3%)	1.75 (0.61, 4.98)	0%	0.30
Without exercise	3	159	11 of 95 (11.6%)	9 of 64 (14.1%)	0.96 (0.44, 2.08)	0%	0.92
Design double-blind design	3	159	11 of 95 (11.6%)	9 of 64 (14.1%)	0.96 (0.44, 2.08)	0%	0.92
No double-blind design	2	162	9 of 82 (11.0%)	5 of 80 (6.3%)	1.75 (0.61, 4.98)	0%	0.30
Population specific ICU patients	2	199	15 of 101 (14.9%)	11 of 98 (11.2%)	1.31 (0.65, 2.63)	0%	0.45
Mixed ICU patients	3	122	5 of 76 (6.6%)	3 of 46 (6.5%)	0.98 (0.26, 3.77)	0%	0.98
Lntake route tube-feeding	3	159	12 of 95 (12.6%)	10 of 64 (15.6%)	0.96 (0.46, 2.00)	0%	0.91
Tube-feeding/oral	2	162	8 of 82 (9.8%)	4 of 80 (5.0%)	1.96 (0.62, 6.25)	0%	0.26
Location Asian study	2	162	9 of 82 (11.0%)	5 of 80 (6.3%)	1.75 (0.61, 4.98)	0%	0.30
Non-Asian study	3	159	11 of 95 (11.6%)	9 of 64 (14.1%)	0.96 (0.44, 2.08)	0%	0.92

ICU, intensive care unit; NG, nasogastric tube; NJ, nasojejunal tube; PEG, percutaneous gastrostomy; RCT, randomized controlled trials.

### Secondary outcomes

**FIGURE 3:**
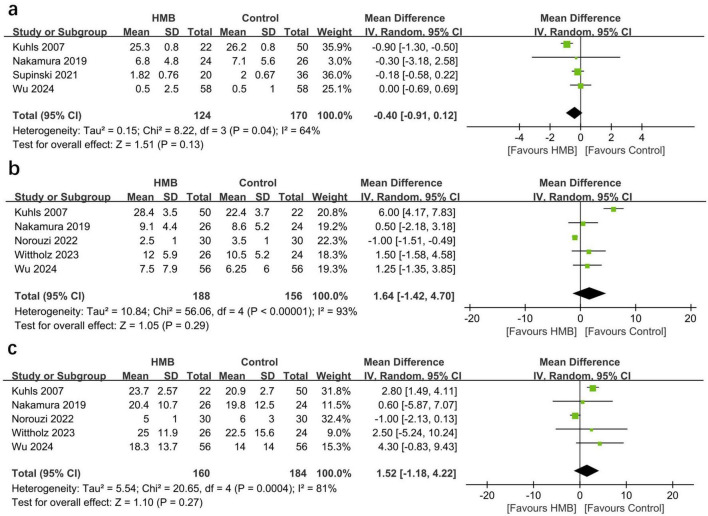
Forest plots of the p beta-hydroxy-beta-methylbutyrate on duration of mechanical ventilation **(A)**, length of stay in intensive care unit (ICU) **(B)**, and length of stay in hospital **(C)** in critically ill patients.

The pooled estimates showed that compared with the control group, the HMB group showed no differences in the duration of MV (MD = −0.40; 95% CI, −0.91 to 0.12; *I*^2^ = 64%; *P* = 0.13; Figure 3A) (20, 29, 31, 32), length of ICU stay (MD = −0.61 days; 95% CI, −3.59 to 2.38; *I*^2^ = 93%; *P* = 0.69; Figure 3B) (20, 22, 29, 30, 32), and length of hospital stay (MD = 1.52 days; 95% CI, −1.18 to 4.22; *I*^2^ = 81%; *P* = 0.27; Figure 3C) (20, 22, 29, 30, 32).

Three studies reported changes in body weight/body mass index after treatment. The pooled results showed that HMB did not affect the changes in body weight (MD = −0.47 kg; 95% CI, −1.96 to 1.01; *I*^2^ = 0%; *P* = 0.53) or body mass index (MD = 0.21 kg/m^2^; 95% CI, −0.48 to 0.89; *I*^2^ = 0%; *P* = 0.56). Two studies described changes in quadriceps thickness and suggested no differences between the HMB and control groups (MD = 0.52; 95% CI, −2.61 to 3.56; *I*^2^ = 95%; *P* = 0.74). The outcomes of changes in skeletal muscle area (*P* = 0.95) and percenters in muscle loss (*P* = 0.16) were similar between the two groups. In addition, only one study by Meng et al. showed that the HMB group had a significantly lower muscle mass change score (*P* < 0.0001) than in the control group.

## Discussion

Our study indicated that muscle loss commonly occurs in critically ill patients. This finding is in line with previous research on sarcopenia in this population. The current meta-analysis of nine RCTs revealed that neither HMB alone nor HMB complexes were associated with improved clinical outcomes in patients in the ICU. Specifically, HMB did not reduce mortality, and MV duration and ICU stay remained the same with HMB administration. Additionally, a few studies have suggested no significant differences in muscle measurements, such as muscle volume or thickness, between the HMB and control groups.

### HMB technology research

Our findings were unexpected, as previous studies have shown that HMB could safely mitigate muscle loss in older adults, cancer patients, and human immunodeficiency virus/acquired immune deficiency syndrome patients (14–16, 33). There is also evidence that HMB complexes can improve muscle strength in patients with postoperative malnutrition or rheumatoid cachexia (18). In addition to muscle measurements, HMB used after discharge in patients with chronic lung disease and heart disease significantly reduced mortality (34). Similarly, in another study, HMB administration to malnourished elderly patients reduced their 90-day mortality and improved their nutritional status (34). The success of HMB in other settings has stimulated several clinical attempts in critically ill patients (18).

However, our meta-analysis did not support the benefits of HMB alone or in combination with its complexes in critically ill patients. Most of the included trials focused on HMB’s effect on different clinical outcomes (20–23, 29–32), including mortality, and indicated no benefits from HMB. In the study by Kuhls et al., HMB resulted in a longer MV duration and ICU and hospital stays (20). Conversely, Norouzid et al. found that perioperative HMB supplementation significantly reduced hospital stay in patients undergoing cardiac surgery (30).

Approximately half of the included trials focused on the effects of HMB on muscle metabolism (19, 21–23, 29, 31). These trials evaluated different muscle metrics and most showed that HMB supplementation during the acute phase of critical care did not prevent muscle loss. Additionally, the included trials suggested that HMB can reduce inflammation and catabolism (21) and improve amino acid metabolism (19), nitrogen balance (20), and nutritional status (19). However, these effects were not due to a reducing in muscle protein turnover rates, which was initially hypothesized. A recent study found that a combination of resistance training and HMB, but not HMB alone, improved physical function and muscle strength in medical ICU patients but had no effect on muscle quality, quality of life, or 60-day mortality (32).

### Interpretation of study results

The negative results of this study may be attributed to several factors. Insufficient study power is a classic explanation for this finding. For example, most RCTs included too few patients to statistically detect the effect of the intervention. However, the negative results can also be due to different HMB strategies in the included trials, not just the statistical conditions. In this situation, increasing the sample size may not have an additional effect. These interventions are heterogeneous for rehabilitation exercises, HMB dosage, timing of outcome assessment, nutritional adequacy, and primary outcome. Exploring this heterogeneity is important because it not only helps us explain our results but also provides insights into future research designs that explore the clinical efficacy of HMB for critically ill patients.

### Timing of administration

Early HMB administration may prevent the development of sepsis-associated muscle dysfunction (10, 35). It blocks the reduction in protein synthesis induced by early sepsis (36). Theoretically, HMB administered after muscle weakness does not improve muscle function. There are several reasons why patients experience muscle weakness in the ICU. The muscle fiber cross-sectional area in ICU patients decreases by 1–2% one day. After seven days, the rectus femoris cross-sectional area decreased by 10.3% (3). Most of the included studies recruited patients on MV (20, 21, 29, 31, 32) or patients with severe trauma (19, 20, 22). This indicated that muscle dysfunction was present before enrollment. For example, in the study by Supinski et al. (31), patients were on MV for an average of 6 days before receiving HMB supplementation. This delay may have hindered the beneficial effects of treatment.

### HMB administration: dosage and route

HMB was administrated at a dose of 3g/day in all included studies. This standard dose has been used in many previous studies of non-critically ill populations (14–16). It has shown beneficial effects on muscle function and tolerability without significant side effects (14–18). However, it is unclear whether the total dose of HMB prescribed for critically ill patients is appropriate. In this population, gastrointestinal dysfunction, fasting, and gastric decompression are frequent, which may impair drug absorption and limit the effectiveness of HMB in improving muscle function (37). To further evaluate the role of HMB, dose-response trials are required.

### Early rehabilitation

Three studies reported on early rehabilitation (29, 31, 32). One of these studies reported inadequate training (<10 min/day) (31). In the study by Wu et al. (32), resistance training (RT) was conducted throughout the hospitalization of medical ICU patients from ICU admission to discharge. This could explain the significant improvements in physical function, muscle strength, and physical activity observed in the RT and HMB + RT groups. However, HMB alone did not produce these effects, highlighting the importance of RT (32). Early rehabilitation is a non-pharmacological intervention that can directly or indirectly support muscle protein turnover (38, 39). It can benefit critically ill patients as it may reduce muscle wasting while enhancing muscle strength (40, 41). HMB alone may have limited effects due to restricted physical activity and insufficient exercise-induced stimulation of muscle protein synthesis (32). In another study focusing on the effects of HMB in ICU patients, the authors suggested that their negative results could be due to the active early rehabilitation applied in both patient groups (29).

### Nutritional adequacy

HMB treatment should be on adequate nutritional therapy. However, only five included studies reported nutritional treatment regimens (20, 22, 23, 29, 32). Only one of these studies used indirect calorimetry for caloric intake (23), whereas the other four relied on weight-based predictive formulas (i.e., 20–30 kcal/kg/d) (20, 22, 29, 32). Thus, these patients may be at risk for over- or under-caloric intake. The protein doses used in these five trials were within the guidelines (i.e., 1.2–2.0 g/kg/d) (20, 22, 23, 29, 32). However, none of these protein regimens have addressed the need for individualized nutrition in critically ill patients. All groups in Kuhls’s study had a negative nitrogen balance during the study period (20). Viana et al. found that the average daily protein intake of both HMB and the control groups fell below 1.2 g/kg at day 4, with 0.90 g/kg/d and 0.82 g/kg/d respectively (23). Therefore, future research should focus on improving the effectiveness of HMB interventions by ensuring nutritional adequacy.

### HMB monotherapy or combined use

Four studies in the current meta-analysis combined HMB and other immune supplements, such as arginine, glutamine, and eicosapentaenoic acid (20, 29–31). It was impossible to confirm that such HMB complexes had any effect owing to the small sample size. Previous studies have shown that arginine and glutamine supplements may aid muscle synthesis and benefit critically ill patients (42, 43). In contrary, in their meta-analysis, Heyland et al. found that arginine supplementation increased mortality in critically ill patients (44). When analyzing studies with higher methodological quality scores, they also found that immune nutrition was associated with a significantly higher mortality rate (44). In one of the included studies, Kuhls reported that the average nitrogen balance for the control group, HMB combined with arginine or glutamine group, and HMB group was −9, −10.9, and −6.5 g/d, respectively (*P* < 0.05) (20). Interestingly, the HMB combination group maintained in a more negative nitrogen balance throughout the study (20). This suggests that the addition of arginine or glutamine to HMB offsets its benefits.

### Limitations

Our study is the first to evaluate HMB in critically ill patients. However, this meta-analysis had some limitations. First, all included trials had a small sample size and may have introduced bias. Second, the HMB strategies varied among the included studies, such as dosage, timing, route, and duration, which might also have prognostic value. However, the original trying to perform subgroup analyses to explore their influences according to such diversities was hindered by insufficient data. Third, only a few trials have reported the effects of HMB on muscles, resulting in a lack of statistical power. This study also failed to measure important metrics such as muscle strength, grip power, and functional status, which are crucial in treating patients in this population. Fourth, these studies used ultrasound rather than the traditional “gold-standard” method for measuring muscles, which requires careful interpretation. Finally, HMB could affect critically ill patients differently, depending on their cause. However, owing to the limited sample size in the existing trials, we could not fully analyze their effects related to specific causes.

## Conclusion

In conclusion, our analysis suggests that HMB alone or in combination did not significantly reduce mortality in critically ill patients. Meanwhile, HMB did not improve the MV duration and length of stay in the ICU or hospital. A few studies included in the analysis suggested that HMB did not improve muscle wasting in patients in the ICU. The limitations of the included studies are prominent, such as the study design and high risk of bias, which may have contributed to the low certainty of our results. Future research should be designed to clarify the effects of HMB in critically ill patients.

## Data Availability

The original contributions presented in this study are included in this article/Supplementary material, further inquiries can be directed to the corresponding author.
