# Supplementary material for: Beta-hydroxy-beta-methyl butyrate supplementation in critically ill patients: a systematic review and meta-analysis of randomized controlled trials
Source: Front Nutr. 2025 Jan 23;12:1505797. doi: 10.3389/fnut.2025.1505797 (PMC11798817; doi:10.3389/fnut.2025.1505797)
Supplement: Supplementary file 1 [file Data_Sheet_1.docx]

**Beta-hydroxy-beta-methylbutyrate supplementation in critically ill patients: a systematic review and meta-analysis of randomized controlled trials**

**Additional files**

Additional file 1 PRISMA checklist……………………………………………………………………………………………………………………………………………………………………………………………….……………………………………..2

Additional file 2 Search Strategy………………………………………………………………………………………………………………………………………………………………………………………………………………….……………………..5

Additional file 3 Summarizations of HMB strategies in the included studies………………………………………………………………………………………………………………………………………….……………………..7 Additional file 4 Assessment of RCTs quality …………….…………………………….…………………………………………………………………………………………………………………………………………………………….....….…….7

Additional file 5 Funnel plot of comparison: short-term mortality…..…………………………………………………………………….…………………………………………………………….…………………………………....….……8

Additional file 6 GRADE evaluation…………………………. …………….…………………………….………………………………………………………………………………………………………………………………………………....….…….9

**Additional File 1**

**PRISMA 2009 checklist**

| **Section/topic** | **#** | **Checklist item** | **Reported on page #** |
| --- | --- | --- | --- |
| **TITLE** | | |  |
| Title | 1 | Identify the report as a systematic review, meta-analysis, or both. | 1 |
| **ABSTRACT** | | |  |
| Structured summary | 2 | Provide a structured summary including, as applicable: background; objectives; data sources; study eligibility criteria, participants, and interventions; study appraisal and synthesis methods; results; limitations; conclusions and implications of key findings; systematic review registration number. | 3 |
| **INTRODUCTION** | | |  |
| Rationale | 3 | Describe the rationale for the review in the context of what is already known. | 5 |
| Objectives | 4 | Provide an explicit statement of questions being addressed with reference to participants, interventions, comparisons, outcomes, and study design (PICOS). | 5-6 |
| **METHODS** | | |  |
| Protocol and registration | 5 | Indicate if a review protocol exists, if and where it can be accessed (e.g., Web address), and, if available, provide registration information including registration number. | 7 |
| Eligibility criteria | 6 | Specify study characteristics (e.g., PICOS, length of follow-up) and report characteristics (e.g., years considered, language, publication status) used as criteria for eligibility, giving rationale. | 7 |
| Information sources | 7 | Describe all information sources (e.g., databases with dates of coverage, contact with study authors to identify additional studies) in the search and date last searched. | 7 |
| Search | 8 | Present full electronic search strategy for at least one database, including any limits used, such that it could be repeated. | 7-8 and Additional File 2 |
| Study selection | 9 | State the process for selecting studies (i.e., screening, eligibility, included in systematic review, and, if applicable, included in the meta-analysis). | 7 |
| Data collection process | 10 | Describe method of data extraction from reports (e.g., piloted forms, independently, in duplicate) and any processes for obtaining and confirming data from investigators. | 7-8 |
| Data items | 11 | List and define all variables for which data were sought (e.g., PICOS, funding sources) and any assumptions and simplifications made. | 8 |
| Risk of bias in individual studies | 12 | Describe methods used for assessing risk of bias of individual studies (including specification of whether this was done at the study or outcome level), and how this information is to be used in any data synthesis. | 8 |
| Summary measures | 13 | State the principal summary measures (e.g., risk ratio, difference in means). | 8 |
| Synthesis of results | 14 | Describe the methods of handling data and combining results of studies, if done, including measures of consistency (e.g., I^2^) for each meta-analysis. | 9 |

| Risk of bias across studies | 15 | Specify any assessment of risk of bias that may affect the cumulative evidence (e.g., publication bias, selective reporting within studies). | 8 |
| --- | --- | --- | --- |
| Additional analyses | 16 | Describe methods of additional analyses (e.g., sensitivity or subgroup analyses, meta-regression), if done, indicating which were pre-specified. | 9 |
| **RESULTS** | | |  |
| Study selection | 17 | Give numbers of studies screened, assessed for eligibility, and included in the review, with reasons for exclusions at each stage, ideally with a flow diagram. | 10and Figure 1, Additional File 2 |
| Study characteristics | 18 | For each study, present characteristics for which data were extracted (e.g., study size, PICOS, follow-up period) and provide the citations. | 10  Table 1, |
| Risk of bias within studies | 19 | Present data on risk of bias of each study and, if available, any outcome level assessment (see item 12). | 10-11 |
| Results of individual studies | 20 | For all outcomes considered (benefits or harms), present, for each study: (a) simple summary data for each intervention group (b) effect estimates and confidence intervals, ideally with a forest plot. | 11 |
| Synthesis of results | 21 | Present results of each meta-analysis done, including confidence intervals and measures of consistency. | 11-12 |
| Risk of bias across studies | 22 | Present results of any assessment of risk of bias across studies (see Item 15). | Additional File 3 |
| Additional analysis | 23 | Give results of additional analyses, if done (e.g., sensitivity or subgroup analyses, meta-regression [see Item 16]). | 11-12  Additional File 4-5 |
| **DISCUSSION** | | |  |
| Summary of evidence | 24 | Summarize the main findings including the strength of evidence for each main outcome; consider their relevance to key groups (e.g., healthcare providers, users, and policy makers). | 13-17 |
| Limitations | 25 | Discuss limitations at study and outcome level (e.g., risk of bias), and at review-level (e.g., incomplete retrieval of identified research, reporting bias). | 18 |
| Conclusions | 26 | Provide a general interpretation of the results in the context of other evidence, and implications for future research. | 19 |
| **FUNDING** | | |  |
| Funding | 27 | Describe sources of funding for the systematic review and other support (e.g., supply of data); role of funders for the systematic review. | 20 |

**Additional File 2**

**Search Strategy**

Database: 5 data bases

Search completed May 1st, 2024

----------------------------------------------------------------------------------------------------------------------

**PubMed**

1#.

(("Critical Care"[Mesh]) OR ((((critical care[Title/Abstract]) OR (critically ill[Title/Abstract])) OR (intensive care[Title/Abstract])) OR (((((((((((((((Critical Illness[Title/Abstract]) OR (Critical Care[Title/Abstract])) OR (intensive care units[Title/Abstract])) OR (Burn units[Title/Abstract])) OR (coronary care units[Title/Abstract])) OR (respiration, artificial[Title/Abstract])) ) OR (ventilators, mechanical[Title/Abstract])) OR (pulmonary ventilation[Title/Abstract])) OR (respiratory insufficiency[Title/Abstract])) OR (multiple organ failure[Title/Abstract])) OR (systemic inflammatory response syndrome[Title/Abstract])) OR (respiratory distress syndrome, adult[Title/Abstract])) OR (sepsis[Title/Abstract])) OR (shock, septic[Title/Abstract]))))

2#.

((Beta-hydroxy-beta-methyl butyrate[Title/Abstract]) OR (hydroxy methylbutyrate[Title/Abstract])) OR (HMB[Title/Abstract])

3.

(randomized controlled trial [pt] OR controlled clinical trial [pt] OR randomized [tiab] OR placebo [tiab] OR clinical trials as topic [mesh: noexp] OR randomly [tiab] OR trial [ti]) NOT (animals [mh] NOT humans [mh])

4.

1# AND 2# AND 3#

**Embase**

No. Query

#21. #12 AND #16 AND #20

#20. #17 OR #18 OR #19

#19. random*:ab,ti AND [embase]/lim

#18. blind*:ab,ti AND [embase]/lim

#17. placebo:ab,ti AND [embase]/lim

#16. #13 OR #14 OR #15

#15. 'HMB':ab,ti AND [embase]/lim

#14 'hydroxy methylbutyrate':ab,ti AND [embase]/lim

#13. 'Beta-hydroxy-beta-methyl butyrate':ab,ti AND [embase]/lim

#12. #1 OR #2 OR #3 OR #4 OR #5 OR #6 OR #7 OR #8 OR #9 OR #10 OR #11

#11. 'sepsis':ab,ti AND [embase]/lim

#10. 'wound':ab,ti AND [embase]/lim

#9. 'trauma':ab,ti AND [embase]/lim

#8. 'icu':ab,ti AND [embase]/lim

#7. 'critical illness':ab,ti AND [embase]/lim

#6. 'intensive care':ab,ti AND [embase]/lim

#5. 'critically ill':ab,ti AND [embase]/lim

#4. 'critical care':ab,ti AND [embase]/lim

#3. 'septic shock':ab,ti AND [embase]/lim

#2. 'bacteremia':ab,ti AND [embase]/lim

#1. 'intensive care'/exp

**Cochrane library**

ID Search

#1 ("intensive care"):ti,ab,kw (Word variations have been searched)

#2 ("critically ill"):ti,ab,kw (Word variations have been searched)

#3 ("critical care"):ti,ab,kw (Word variations have been searched)

#4 ("critical illness"):ti,ab,kw (Word variations have been searched)

#5 ("Burn"):ti,ab,kw (Word variations have been searched)

#6 ("acute respiratory distress syndrom"):ti,ab,kw (Word variations have been searched)

#7 ("truma"):ti,ab,kw (Word variations have been searched)

#8 ("septic shock"):ti,ab,kw (Word variations have been searched)

#9 #1 OR #2 OR #3 OR #4 OR #5 OR #6 OR #7 OR #8

#10 (pectin):ti,ab,kw (Word variations have been searched)

#11 #9 AND #10

**Wanfang database**

主题：（危重症 or 重症监护or 重症 or ICU or 脓毒症 or 创伤）and 主题：（β-羟基-β-甲基丁酸盐）

**China National Knowledge Infrastructure database**

TKA=“β-羟基-β-甲基丁酸盐” and TKA=("危重症 + “重症监护” + “重症” + “ICU” + “脓毒症” + “创伤”）

**Additional File 3**

**HMB strategies of the included RCTs**

| **Study** | **Sample size** | **Timing of HMB administration** | **Dosage and route of HMB** | **Early rehabilitation** | **Nutritional protocols** | **HMB monotherapy or combined use** |
| --- | --- | --- | --- | --- | --- | --- |
| Hsieh 2006 [20] | 34 | NA | HMB calcium salt 3g/d for 7 days, in 2 equal daily doses through nasogastric feeding | NA | Mixed commercial formulas: Calories: 1330 kcal/L, 30% from carbohydrate, 47% from fat, 23% from protein | HMB monotherapy |
| Kuhls 2007 [19] | 100 | NA | 3 g HMB calcium salt for 28 days through tube feeding | NA | Calories: 25 kcal//kg/d;  Protein: 1.5 g/kg/day | HMB monotherapy: 3 g;  Combined Use: HMB + 14 g L-arginine + 14 g L-glutamine |
| Meng 2021 [18] | 56 | NA | 3 g/day: HMB 1.5 g twice daily for 3 months through tube feeding | NA | NA | HMB monotherapy |
| Nakamura 2019 [28] | 88 | Day 2 | 3 g/day: 1.5 g HMB Ca twice a day until day 10 through tube feeding | EMS 20 min at a time, Other PT in other than the 20-minute intervention. | Calories: 20-30 kcal/kg/d | Combined use: 3 g HMB,+14 g arginine, + 14 g glutamine daily |
| Norouzi 2022 [29] | 70 | 30 days before cardiac surgery | 3 g daily (2 sachets/day of a combination of1.5 g HMB) for 1 months through oral | NA | NA | Combined use: 7 g L -arginine + 7 g L -glutamine + 1.5 g HMB |
| Supinski 2021 [30] | 83 | NA | HMB: 3 g/day, (1500 mg HMB every 12h) through tube feeding  HMB + EPA: 3 g/day (1500 mg HMB every 12 h) through tube feeding | Three PT sessions per week, 20-40 min at a time | Calories: basal energy expenditure was estimated based on the Harris- Benedict equations.  Protein: unstressed: 0.8-1.0 gm/kg, mild stress: 1.0-1.2 gm/kg, moderate stress: 1.2-1.5 gm/kg, infection or severe stress: 1.3-1.6 gm/kg | HMB monotherapy: 1500 mg HMB,  Combined use: 1500 mg HMB and 1000 mg EPA |
| Viana 2021 [22] | 37 | Day 4 | 3g/day through tube feeding | NA | Calories: Initial 20 kcal//kg/d, From day 4: indirect calorimetry,  protein: 1.2 -1.3 g/kg/day | HMB monotherapy |
| Wittholz 2023 [21] | 33 | At randomization | 3g through tube feeding or oral | NA | Calories: 25 kcal/kg of IBW, >7 days and do not meet contraindications: measure MEE using indirect calorimetry;  Protein: 1.2–2.0 g/kg/day | HMB monotherapy |
| Wu 2023 [31] | 112 | With enteral nutrition | 3.0g/day (1.5 g HMG Ca twice daily) through tube feeding or oral | Five times per week, each session 20-30 min | Calories: the acute phase: 20-25 kcal/kg per day, the stable phase: 25–30 kcal/kg per day;  Protein: 1.2-2 g/kg per day | HMB monotherapy |

EMS = electrical muscle stimulation; EPA = eicosapentaenoic acid; Harris- Benedict equations: BEE = base energy expenditure; HMB = beta -hydroxy -beta -methylbutyrate; IBW = ideal body weight, MEE = metabolic energy expenditure; PT = physical therapy; RT = resistance training.

**Additional File 4**

**Assessment of study quality**


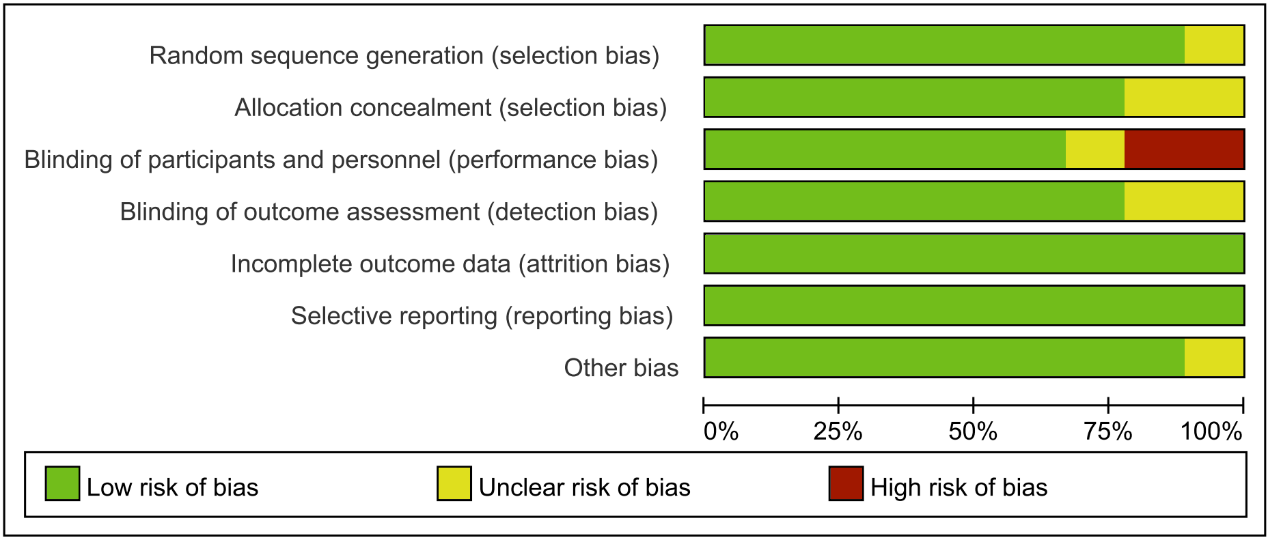


**Figure S1. Risk of bias graph: review authors' judgements about each risk of bias item presented as percentages across all included studies.**


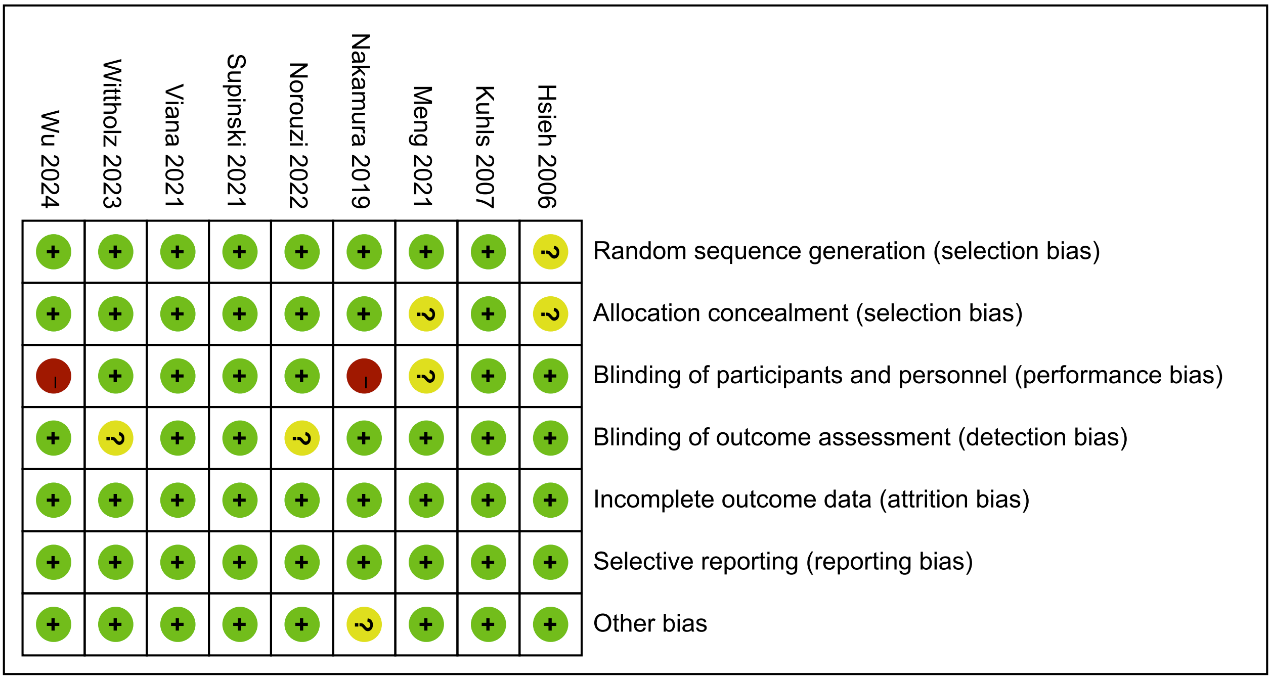


**Figure S2. Risk of bias summary: review authors' judgements about each risk of bias item for each included study**

**Additional File 5**

**Funnel plot of comparison: Mortality**


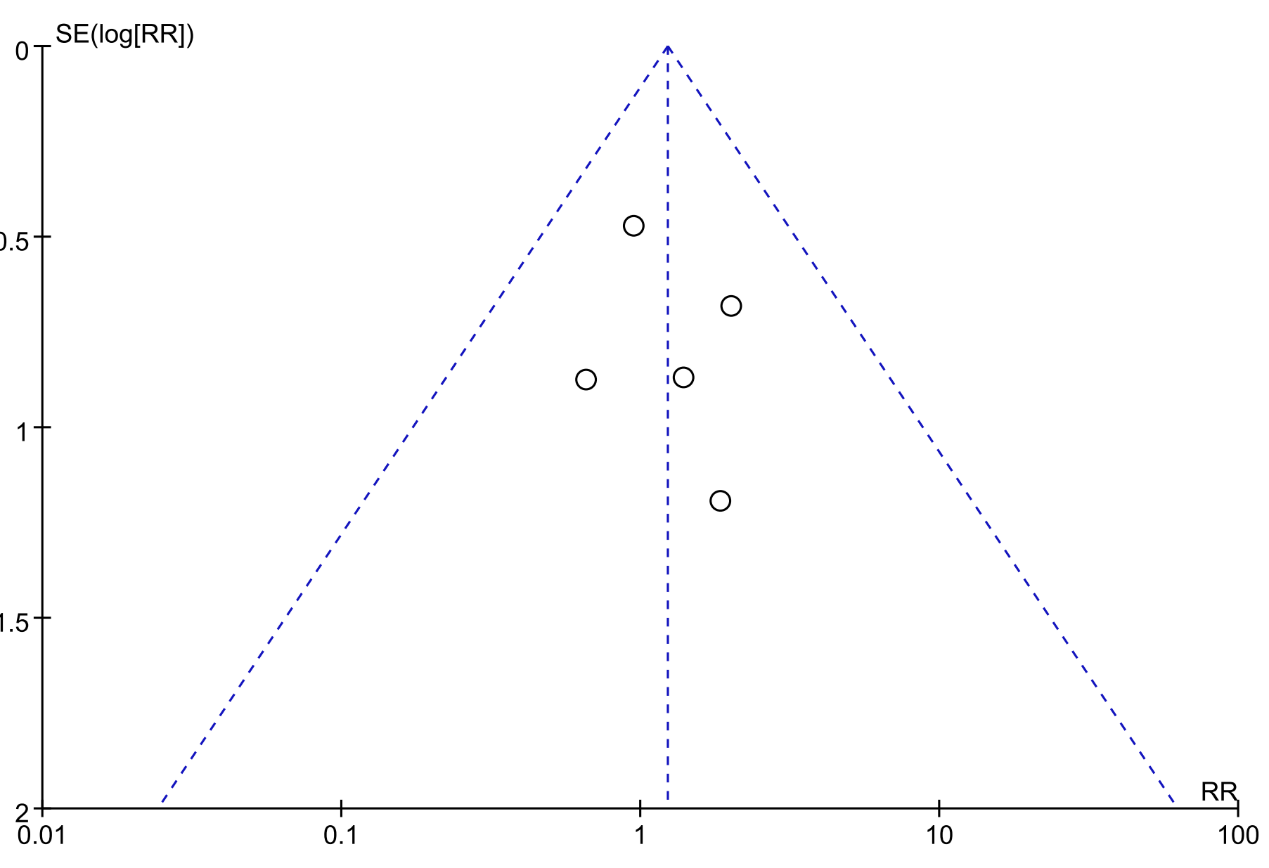


**Additional File 6: GRADE** **profile.**


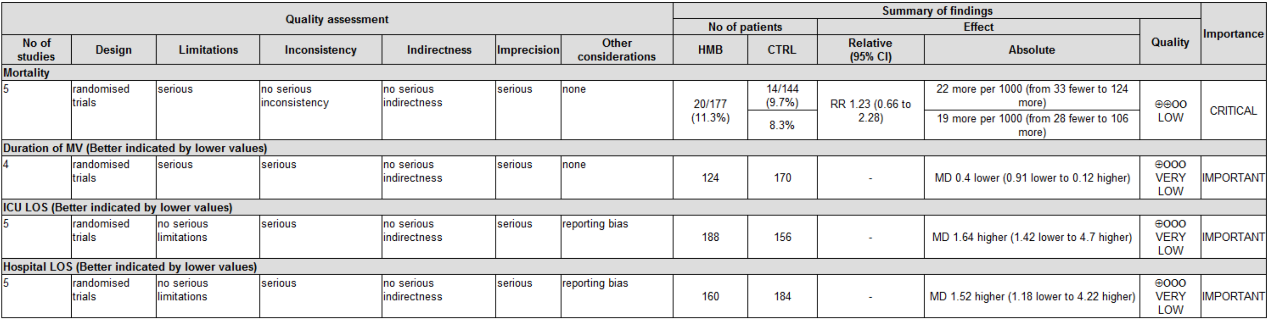


GRADE Working Group grades of evidence

- High quality: Further research is very unlikely to change our confidence in the estimate of effect.

- Moderate quality: Further research is likely to have an important impact on our confidence in the estimate of effect and may change the estimate.

- Low quality: Further research is very likely to have an important impact on our confidence in the estimate of effect and is likely to change the estimate.

- Very low quality: We are very uncertain about the estimate.
